# Repeat exit site infection in peritoneal dialysis patient with polycythemia vera – a case report

**DOI:** 10.1186/s12879-021-06342-x

**Published:** 2021-06-30

**Authors:** Edyta Gołembiewska, Kazimierz Ciechanowski

**Affiliations:** Department of Nephrology, Transplantology and Internal Medicine, Pomeranian Medical University, Al. Powstańców Wielkopolskich 72, 70-111 Szczecin, Poland

**Keywords:** Case report, Exit site infection, Peritoneal dialysis, Polycythemia vera

## Abstract

**Background:**

Infectious complications of peritoneal dialysis (PD) remain a common cause of catheter loss and discontinuation of PD. Exit site infection (ESI) constitutes a significant risk factor for PD-related peritonitis and determination of predisposing states is relevant. We here present a case of repeat ESI due to *Pseudomonas aeruginosa* in a PD patient with skin changes in the course of polycythemia vera (PV).

**Case presentation:**

A 73-year-old PD patient with chronic kidney disease secondary to renal amyloidosis and ankylosing spondylitis, presented to the nephrology unit with signs of ESI. In 2006 he was diagnosed with PV and since then has was successfully treated with hydroxyurea; however, he reported recurrent episodes of developing skin nodules in the course of the disease. Exit site swab yielded *Pseudomonas aeruginosa* and the infection developed in the ulcerated PV nodule that appeared in exit site 2 weeks earlier. Patient was treated with intraperitoneal amikacin and oral ciprofloxacin, however, due to neurological complications, the treatment had to be interrupted and finally catheter was removed. Similar episode of ESI with *Pseudomonas aeruginosa* developed in the patient two years earlier and also required catheter removal.

**Conclusion:**

This is the first case report demonstrating the development of ESI on the polycythemia vera skin lesion in this area. Skin manifestations of PV might be a predisposing factor to ESI in PD patients.

## Background

Infectious complications of peritoneal dialysis (PD) include exit site infection (ESI), tunnel infection (TI) and peritoneal dialysis-related peritonitis (PD-related peritonitis) and remain a common cause of catheter loss and discontinuation of PD. ESI constitutes a significant risk factor for peritonitis and determination of predisposing states is relevant [1, 2]. Most ESIs are caused by skin flora, however, resilient microorganisms such as *Pseudomonas spp*. play a significant role in the etiology of this condition. Thus, prevention and appropriate management of ESI is of critical importance in improving patient outcomes. Here, we present a case of repeat ESI caused by *Pseudomonas aeruginosa* in a PD patient with polycythemia vera (PV).

## Case presentation

**Fig. 1:**
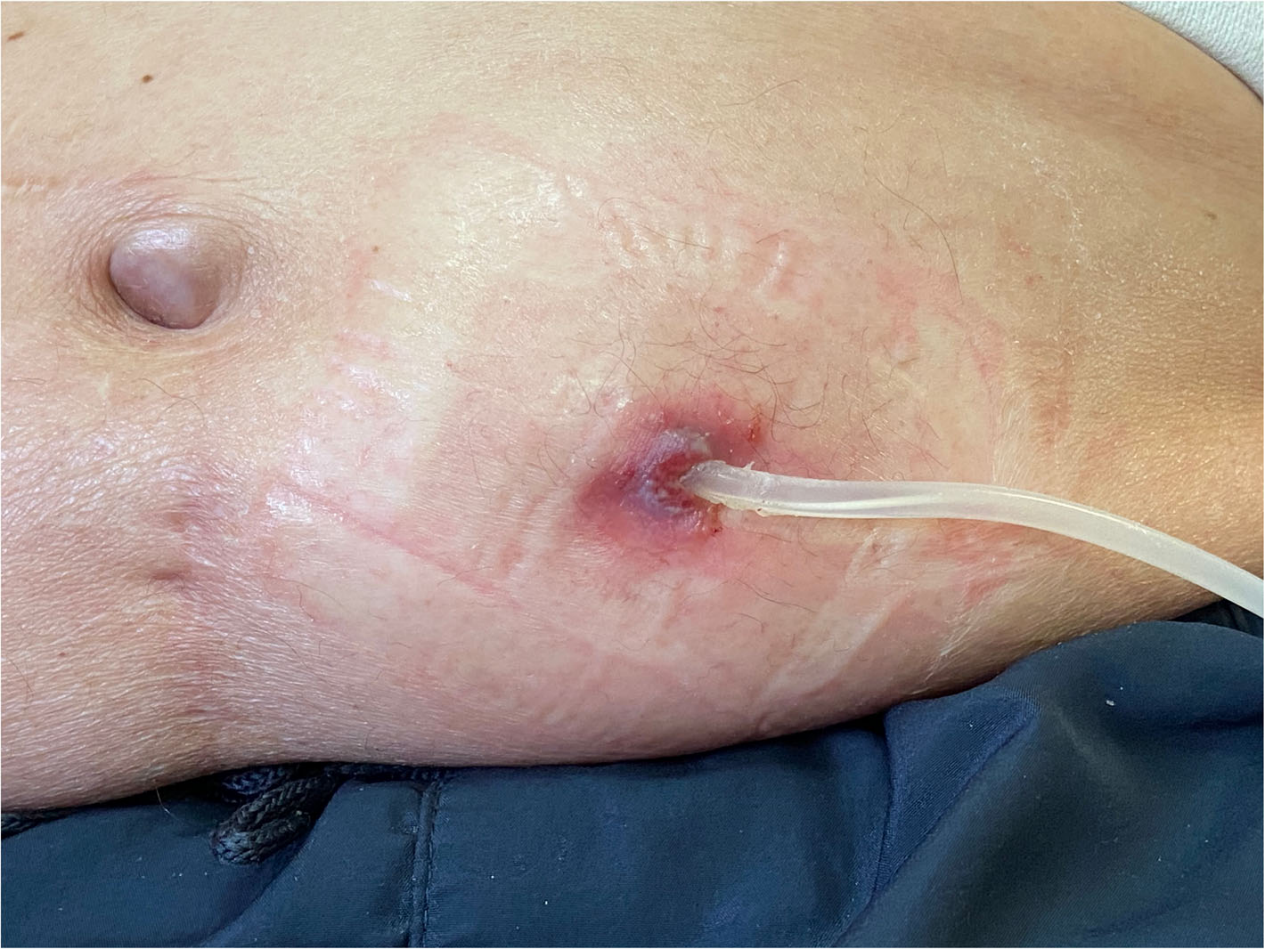
Signs of exit site infection

A 73-year-old peritoneal dialysis patient with PV presented to the nephrology unit with signs of ESI. He complained of pericatheter redness, tenderness as well as bloody and purulent discharge in the area of exit site (ES). In 2016 he started continuous ambulatory peritoneal dialysis (CAPD) with four 2 L exchanges of 1.5% glucose fluid as renal replacement therapy for end-stage renal disease due to renal amyloidosis. This condition developed as a complication of ankylosing spondylitis diagnosed 33 years earlier. PV was diagnosed in 2006 and since the diagnosis the patient was successfully treated with hydroxyurea. The disease remained stable, however, the patient complained of recurring episodes of skin papulas and subcutaneous nodules of red colour. Some of these skin lesions developed ulcerations. The patient reported that episodes of these skin manifestations of PV used to appear with frequency of once per year. He also reported that he had noticed the development of skin changes 2 weeks before the symptoms of ESI, one of the lesions developed in exit site. Later he noticed ulceration of the lesion and purulent discharge in this area (Fig. 1). Swab was taken which yielded *Pseudomonas aeruginosa*. There were no signs of peritonitis and white cell count in dialysis effluent was 8/μL. Patient’s general condition was good, with body temperature 36.5C. His blood pressure was 124/74 mmHg, his heart rate was 72 beats per minute. White blood cell count was within normal limits (7.3 × 10^9^ cells/L), C-reactive protein (CRP) was 24 mg/L. Topical gentamicin 1% cream was used to exit site and treatment with intraperitoneal amikacin and oral ciprofloxacin was started, however, after 10 days of therapy patient complained of severe vertigo and hearing loss. Computed tomography of the brain revealed no pathological changes and neurological signs were attributed to aminoglycoside toxicity. Treatment was reduced to ciprofloxacin, but because of maintaining signs of ESI the decision was taken to transfer the patient to hemodialysis treatment and to remove the peritoneal catheter. The neurological symptoms disappeared within a few days after discontinuation of aminoglycoside therapy. Patient has been undergoing chronic hemodialysis treatment for 9 months now and presents no complaints. Similar episode of ESI with *Pseudomonas aeruginosa* that started with the development of nodule in ES area developed in the patient two years earlier. That episode of ESI was treated with intraperitoneal ceftazidime and oral ciprofloxacin but was also complicated by cuff protrusion what finally led to catheter removal and subsequent implantation of a new Tenckhoff catheter on the other side of abdomen.

## Discussion and conclusions

This case calls attention to skin manifestations of PV as possible predisposing factor to the development of ESI. It also emphasizes that not only peritoneal dialysis-related peritonitis but catheter-related infections lead to technique failure and transfer to hemodialysis.

Cutaneous lesions such as nodules and papules have been rarely reported in patients with PV and involve mainly face, chest or limb area. The appearance of pustules and nodules is usually associated with progressive disease and poor prognosis [3, 4]. In our patient the disease remained stable with recurring episodes of development of skin nodules with some of them undergoing subsequently eruption and involution leaving superficial scars. The development of small skin nodules in our patient can be explained by microvascular and vasomotor complications with arteriolar inflammation and thrombotic occlusions rather than the development of subcutaneous extramedullary hematopoiesis suggesting the possibility of progression of PV to myelofibrosis. Cytokine excess and predisposition to systemic inflammation has also been reported as an important factor explaining many of PV-associated symptoms [4]. In the case of presented patient, apart from inflammation generated by PV, his comorbid disease – ankylosing spondylitis – presents another inflammatory disease which might have influenced experienced symptoms.

ESI is an infectious complication of PD and a known risk factor for subsequent tunnel infection and peritonitis. Therefore, prevention and treatment of ESI is crucial for the long-term outcome of PD patients. The rates of ESI are reported to be within the range of 0.06–0.42 episodes per year. Risk factors of ESI include poor competency of ES care, poor catheter immobilization, history of catheter-pulling injury and mechanical stress on ES [1]. This strengthens the importance of methods of exit site care as well as proper teaching and PD training. Recent study of a standardized education training programme for both PD trainers and PD patients has demonstrated feasibility of implementation in a renal clinical setting [5]. Also, the catheter should be tightly anchored and immobilized. In addition, patients should be educated how to avoid mechanical stress on PD catheter while using their waist belts or protective bag of catheter. In our case, the ulceration of the nodule in the close area of ES might have led to the development of infection as the skin barrier was lost what made an easier way for pathogen invasion and its spreading.

Coagulase-negative staphylococci, Gram positive rods and *Staphylococcus aureus* are the most common pathogens responsible for ESI. However, Pseudomonas spp. can be isolated even in 10% of all ESIs and such infection is associated with high risk of catheter loss due to refractory, recurrent or repeat ESI or peritonitis due to ability of *Pseudomonas spp*. to form biofilm [6, 7]. Therefore, early detection of Pseudomonas ESI is very important and prompt aggressive treatment might prevent the invasion of external cuff, catheter tunnel and finally the development of peritonitis.

The reported cure rate of ESI caused by Pseudomonas and treated with different systemic antibiotic regimens ranges from 38 to 83% [7]. *International Society of Peritoneal Dialysis* (ISPD) recommends that ESIs caused by Pseudomonas species require prolonged therapy with two antibiotics. Oral fluoroquinolones are suggested as first-line therapy, however, these should not be given in monotherapy due to rapidly developing resistance. In cases of recurrent Pseudomonas ESI, ISPD recommends adding intraperitoneal aminoglycoside or ceftazidime [8]. In our patient, treatment with aminoglycoside had to be discontinued after 10 days because of toxic side effects. Little is known about the pharmacokinetics of amikacin in CAPD patients what makes the appropriate dosing in the treatment of ESI or PD-related peritonitis difficult. The implementation of therapeutic drug monitoring may be useful in controlling amikacin serum concentration and prevention of its side-effects [9]. In our patient, as ESI did not resolve, peritoneal catheter was removed and patient was transferred to hemodialysis.

This case is unique as this is the first report of the repeated development of ESI following the episode of the appearance of skin manifestation of PV in the area of ES. The presence of nodular and pustular lesions in the ES might be a predisposing factor to infectious complications of PD.

## Data Availability

All relevant data are shown in the case report.

## Abbreviations

CAPD: continuous ambulatory peritoneal dialysis
CRP: C-reactive protein
ES: exit site
ESI: exit site infection
ISPD: International Society of Peritoneal Dialysis
PD: peritoneal dialysis
PV: polycythemia vera
TI: tunnel infection
